# Human Endometrial Carcinogenesis Is Associated with Significant Reduction in Long Non-Coding RNA, TERRA

**DOI:** 10.3390/ijms21228686

**Published:** 2020-11-18

**Authors:** Meera Adishesh, Rafah Alnafakh, Duncan M. Baird, Rhiannon E. Jones, Shannon Simon, Lucy Button, Areege M. Kamal, John Kirwan, S. Bridget DeCruze, Josephine Drury, Gabriele Saretzki, Dharani K. Hapangama

**Affiliations:** 1 Liverpool Women’s Hospital NHS Foundation Trust, Liverpool L8 7SS, UK; meeadish@liverpool.ac.uk (M.A.); John.Kirwan@lwh.nhs.uk (J.K.); shandyabdecruze@gmail.com (S.B.D.); 2 Department of Women’s and Children’s Health, Institute of Life Course and Medical Sciences, University of Liverpool, Member of Liverpool Health Partners, Liverpool L8 7SS, UK; R.A.A.Alnafakh@liverpool.ac.uk (R.A.); shannonsimon96@gmail.com (S.S.); lucyfbutton@gmail.com (L.B.); areegekamal@gmail.com (A.M.K.); jadrury@liverpool.ac.uk (J.D.); 3 Division of Cancer and Genetics, Cardiff University, Cardiff CF14 4XN, UK; bairddm@cardiff.ac.uk (D.M.B.); jonesr47@cardiff.ac.uk (R.E.J.); 4 The National Centre for Early Detection of Cancer, Oncology Teaching Hospital, Baghdad Medical City, Baghdad, Iraq; 5 Biosciences Institute, Newcastle University, Newcastle upon Tyne NE4 5PL, UK; gabriele.saretzki@newcastle.ac.uk

**Keywords:** TERRA, long non-coding RNA, telomerase, endometrium, telomeres, telomere length, endometrial cancer

## Abstract

Telomeres are transcribed as long non-coding RNAs called TERRAs (Telomeric repeat containing RNA) that participate in a variety of cellular regulatory functions. High telomerase activity (TA) is associated with endometrial cancer (EC). This study aimed to examine the levels of three TERRAs, transcribed at chromosomes 1q-2q-4q-10q-13q-22q, 16p and 20q in healthy (*n* = 23) and pathological (*n* = 24) human endometrium and to examine their association with cellular proliferation, TA and telomere lengths. EC samples demonstrated significantly reduced levels of TERRAs for Chromosome 16p (Ch-16p) (*p* < 0.002) and Chromosome 20q (Ch-20q) (*p* = 0.0006), when compared with the postmenopausal samples. No significant correlation was found between TERRA levels and TA but both Ch-16p and Ch-20q TERRA levels negatively correlated with the proliferative marker Ki67 (r = −0.35, *p* = 0.03 and r = −0.42, *p* = 0.01 respectively). Evaluation of single telomere length analysis (STELA) at XpYp telomeres demonstrated a significant shortening in EC samples when compared with healthy tissues (*p* = 0.002). We detected TERRAs in healthy human endometrium and observed altered individual TERRA-specific levels in malignant endometrium. The negative correlation of TERRAs with cellular proliferation along with their significant reduction in EC may suggest a role for TERRAs in carcinogenesis and thus future research should explore TERRAs as potential therapeutic targets in EC.

## 1. Introduction

Endometrial cancer (EC) is the most common gynecological malignancy in the western world with an increasing incidence due to the rise in obesity and longevity [1]. This increase in frequency of EC is accompanied by a concomitant rise in cancer-associated mortality, a trend that is expected to continue for the next few decades [2]. This is a contrasting and concerning statistic, compared with many other cancers, where the incidence of new cancers remains stable and cancer-associated mortality is decreasing. Therefore, it poses an urgent need to develop novel preventative and treatment strategies, which require advancement in our current understanding of endometrial carcinogenesis. The mortality rates for high-grade EC are especially high, similar to ovarian cancer. Some women with EC are not suitable for the conventional surgical treatment, hysterectomy [3], and surgical treatment is associated with significant morbidity and mortality in those with co-occurring multi-morbidities and obesity. Biomarkers that would inform us of potential prognostic and therapeutic targets are thus invaluable in developing future strategies to reduce the cancer related morbidity and mortality of EC.

High activity levels of telomerase enzyme are a feature of most carcinomas, with telomere maintenance being its main function. Telomeres are specialized nucleoprotein complexes at the ends of chromosomes that prevent chromosomal ends from being recognized as DNA damage foci that can mount a DNA damage response (DDR) [4]. Telomeres contain a repetitive hexanucleotide sequence TTAGGG bound by six-shelterin proteins, namely telomeric repeat binding factor 1 and 2 (TRF1 and TRF2), Protection of telomeres 1 (POT1), tripeptidyl peptidase 1 (TPP1), Repressor/activator protein 1 (RAP1) and TRF1- and TRF1-interacting protein 2 (TIN2), and ends with single-stranded G-rich overhang [5]. Telomeric DNA is lost with each round of cell division, due to the end replication problem, and, unless the lost DNA is replaced by the action of telomerase, telomeres will shorten in proliferating cells. Critically short telomeres can initiate a DDR and induce apoptotic or cellular senescence pathways with permanent cell cycle arrest [6]. However, specialized cells and cancer cells have sufficiently high telomerase activity (TA) for telomere maintenance and elongation, which prevents senescence and allows unrestrained proliferation [7].

Telomeres were considered to be transcriptionally silent until recently, however telomeric repeat-containing RNA (TERRA) molecules are transcribed by RNA polymerase II from CpG-island containing sub telomeric promotors [8]. TERRA molecules are heterogenous long non-coding RNAs (lncRNA) [9], and they have been identified in vertebrates and in several other species including yeasts and plants [10,11]. Transcription of TERRA has been proposed to assist in telomere replication and thus support chromosome stability [12]. The action of TERRA may be relevant to the telomere of origin or other telomeres in general [13]. Their expression is inversely correlated with the length of the corresponding telomeres [14]. TERRAs are involved in the recruitment of telomerase to telomeres [15] when they remain partly associated with the telomere of origin, establishing an RNA G-quadruplex structure that protects the telomeres [16]. The TERRA containing RNA-DNA hybrids at the telomeric end may also have a physiological role in stalling replication forks, hence allowing DNA repair [17]. Conversely, TERRAs are also proposed to inhibit telomerase function by binding to the *hTERC* and hTERT components of telomerase [18], as well as by forming RNA–DNA hybrids [10]. TERRA levels are cell specific [9] and fluctuate with the cell cycle [19]. Shelterin proteins TRF1 and TRF2 are known to interact with TERRA and TERRAs mediate cell-fate and cellular reprogramming via a TRF1-dependent pathway [20]. Therefore, TERRAs are thought to participate in many essential cellular and telomere/telomerase regulatory functions [14]. A study that examined the predicted features of 18 TERRA loci in human cell confirmed transcripts arising from only 20q and Xp loci to have these expected TERRA features, and subsequently demonstrated that only deletion of the 20q locus to cause a dramatic decrease in TERRA levels [13]. By deleting the 20q-TERRA locus, the authors demonstrated an increase in telomere damage foci and confirmed that TERRA transcripts to be essential for the maintenance of a functional telomere cap.

Human endometrium is a dynamic tissue, which undergoes a repetitive cycle of cell proliferation, differentiation, shedding and regeneration during the reproductive years of a woman’s life. It is regulated by the cyclically secreted ovarian hormones on a monthly basis [21]. Although a somatic tissue, human endometrium expresses dynamic TA levels, and highly proliferative endometrial cells during the estrogen dominant, proliferative phase of the menstrual cycle have high TA levels, while the cessation of the epithelial proliferation under the regulation of progesterone in the secretory phase of the cycle demonstrated lowest TA levels [4,22]. Endometrial epithelium demonstrates distinctively higher TA than the stromal cells, yet conversely shorter relative telomere lengths were reported in the epithelial cells when compared with the stromal cells from the same samples [22]. This observation had been explained as high TA maintaining the short epithelial telomeres beyond the critical length, thus allowing epithelial cells to avoid cell cycle arrest/cellular senescence [4,22]. Therefore, TA is implicated in endometrial epithelial cell proliferation; high TA is a reported feature in the normal proliferative phase endometrium, in benign endometrial proliferative conditions such as endometriosis, and in endometrial carcinogenesis [23,24].

As mentioned above, many authors have already reported the involvement of TA, telomeres and many telomere/telomerase associated proteins and genes in endometrial function including their aberrations in endometrial carcinogenesis [25,26,27,28,29,30]. However, the existence of TERRAs in healthy endometrium or their disease-specific alterations have not yet been elucidated. Considering the intricate relationship between telomere/telomerase biology and endometrial cellular proliferation [22] and carcinogenesis [31], we hypothesized that TERRA levels may also be altered in EC. We therefore sought to examine the expression levels of TERRAs in human endometrium, from both healthy women and those with EC. We utilized TERRA primers that measured collective TERRA levels from various chromosome ends (chromosomes 1q, 2q, 4q, 10q, 13q and 22q) and two further specific TERRAs that are transcribed from chromosomes 16p and 20q.

We also examined the relationship of TERRA levels with epithelial cell proliferative index and steroid hormone receptors immuno-scores assessed using immunohistochemistry, as they are established markers predicting prognosis in EC. We also examined the immunoreactivity of two shelterin proteins known to regulate TERRAs, TRF1 (positive regulator) and TRF2 (negative regulator), in the same EC samples to examine their possible correlation with the corresponding TERRA levels. Finally, we analyzed the differential telomere length distributions using high-resolution single telomere length analysis (STELA) at the chromosome XpYp telomeres (which is representative of the genome-wide telomere length) in the same human endometrial samples.

## 2. Results

### 2.1. TERRA Transcription Is Observed in Normal Human Endometrium and the Levels Are Significantly Reduced in Endometrial Cancer

**Figure 1:**
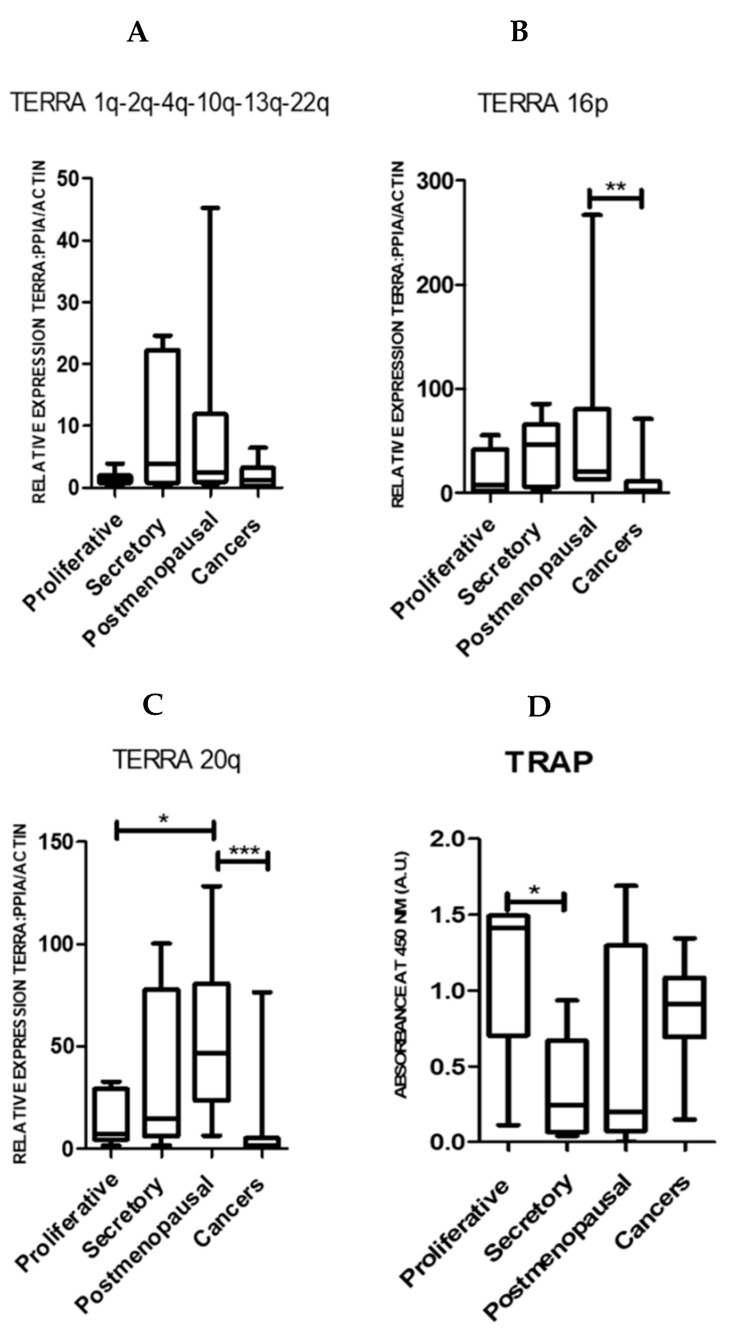
TERRA levels in human endometrium. TERRA levels by qPCR and Telomerase activity by Telomere Repeat Amplification Protocol (TRAP) assay in proliferative (*n* = 6), secretory (*n* = 8), postmenopausal (*n* = 7) human endometrium and endometrial cancers (*n* = 24) for: chromosome 1q-2q-4q-10q-13q-22q (**A**); chromosome 16p (**B**); and chromosome 20q (**C**). When compared to age-matched postmenopausal endometrium (*n* = 7), women with ECs (*n* = 24) showed no changes in TERRA levels for chromosomes 1q-2q-4q-10q-13q-22q (**A**) but a significant decrease in Ch-16p (** *p* = < 0.002), Mann–Whitney-U test (**B**) and Ch-20q (*** *p* = <0.001, * *p* < 0.05); Mann–Whitney-U test (**C**) but no changes in TA (**D**).

Both pre- and postmenopausal endometrium expressed all TERRAs examined (chromosomes 1q-2q-4q-10q-13q-22q, 16p and 20q) (Figure 1A–C). Compared with the proliferative phase samples, the proliferatively quiescent postmenopausal samples had higher levels of Ch-16p and Ch-20q TERRA levels (Figure 1B,C).

Ch-16p (*p* = 0.002) and Ch-20q (*p* = 0.0006) TERRAs were significantly reduced in EC samples when compared with the healthy postmenopausal endometrium (Figure 1B,C). However, TA was high in proliferative phase and EC samples (*p* = 0.19, Figure 1D). In our sample set, the only significant difference in TA was found between the proliferative and secretory phase samples from pre-menopausal women (*p* = 0.04) as previously described [4,32].

**Figure 2:**
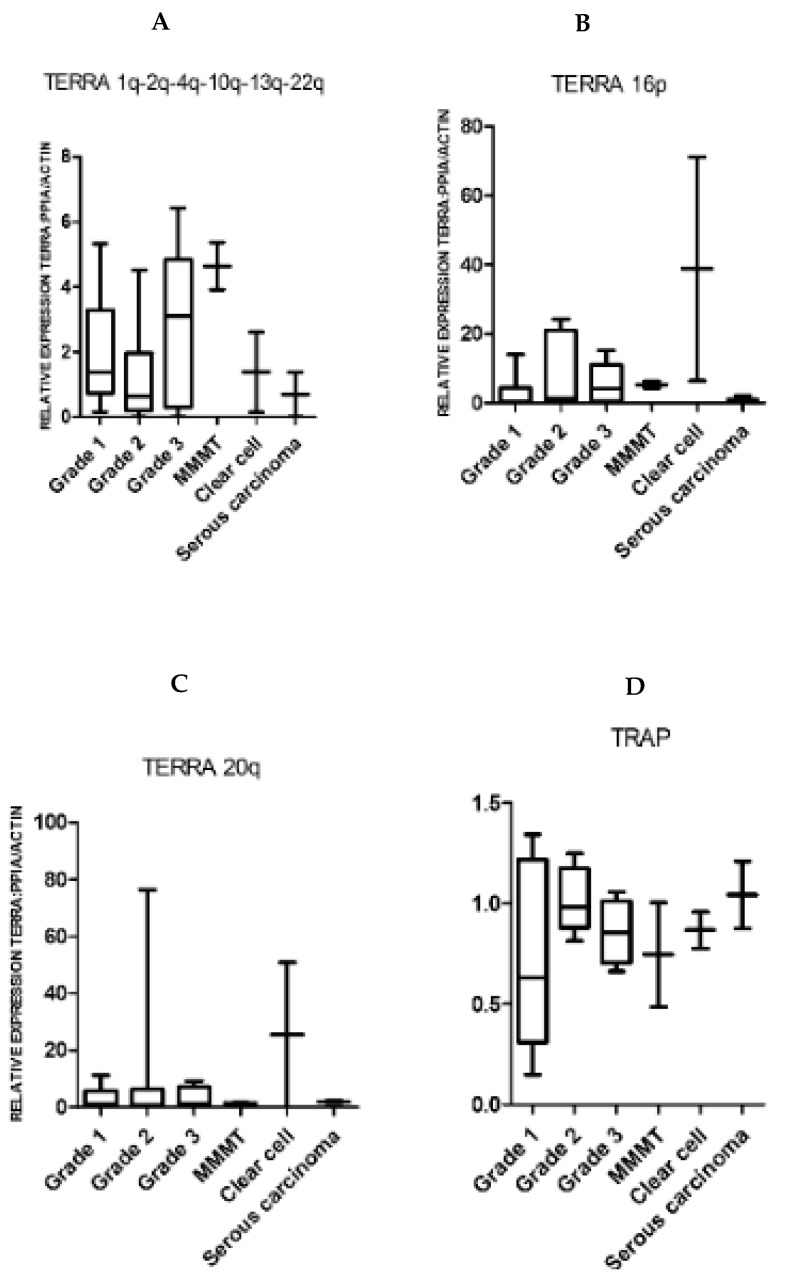
TERRA and TRAP levels in different grades and types of endometrial cancers. Grade 1 adenocarcinoma, *n* = 6; grade 2 adenocarcinoma, *n* = 7; grade 3 adenocarcinoma, *n* = 5; carcinosarcoma/malignant mixed Müllerian tumor (MMMT), *n* = 2; clear cell carcinoma, *n* = 2; serous carcinoma, *n* = 2. Relative normalized expression of chromosomes: 1q-2q-4q-10q-13q-22q (**A**); 16p (**B**); and 20q (**C**). TERRA levels were measured in all types of endometrial cancers. No difference in TERRA levels were found in different grades of endometrioid cancers or individual subtypes of type 2 cancers. All types of endometrial cancers had high telomerase activity as assessed by TRAP assay (**D**).

When the histological grading (as a measure of cellular anaplasia) of EC samples or individual subtypes of type II ECs were considered, neither TERRA levels nor TA changed significantly with the tumor grade/subtype (Figure 2).

### 2.2. Endometrial TERRA Levels Correlated with Each Other, the Proliferative Marker Ki67, Steroid Receptor PR and Shelterin Protein TRF1 but Did Not Correlate with TA or with TLs at the XpYp Chromosomes

**Figure 3:**
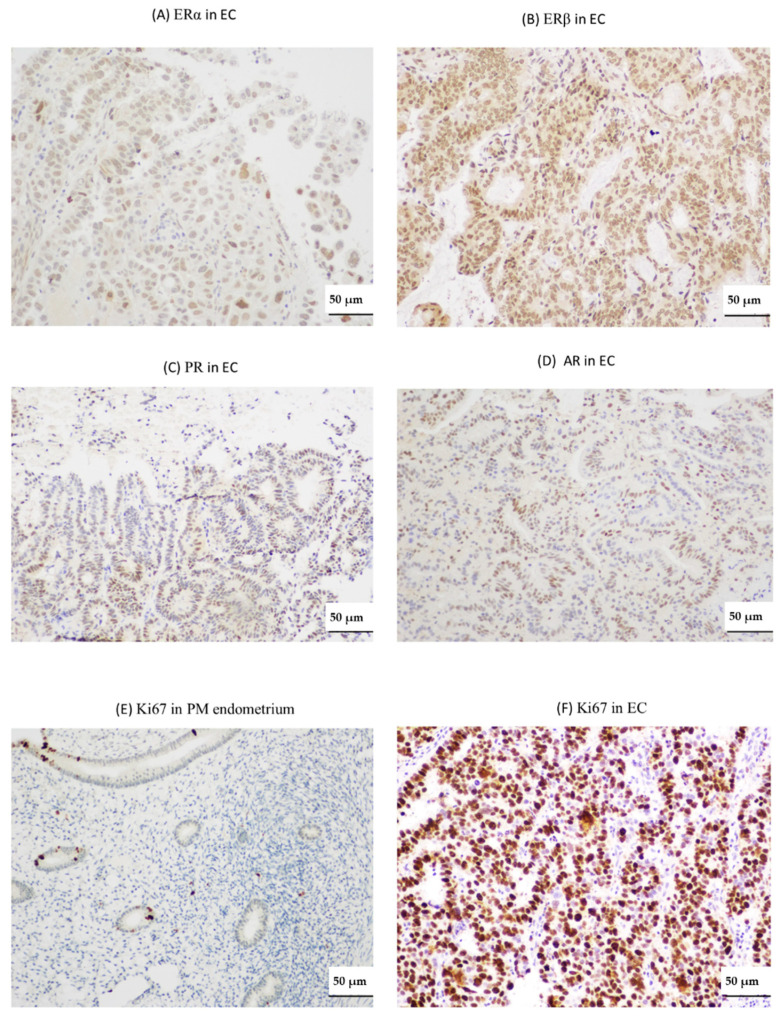
Representative micrographs of steroid receptors and Ki67. Micrograph showing immunostaining in endometrial cancer (EC) of: estrogen receptor α (ERα) (**A**); estrogen receptor β (ERβ) (**B**); progesterone receptor (PR) (**C**); and androgen receptor (AR) (**D**). Micrographs of Ki67 immunostaining: in postmenopausal (PM) endometrium (**E**); and in endometrial cancer (**F**). Scale bar 50 µm, 200× magnification.

Levels of all three TERRAs demonstrated a positive correlation with each other in all endometrial samples (healthy and pathological) examined, suggesting a shared regulation (Table 1: Ch-16p and 20q, r = 0.78, *p* < 0.0001; Ch-16p and Ch-1q-2q-4q-10q-13q-22q, r = 0.49, *p* = 0.001; Ch-20q and Ch-1q-2q-4q-10q-13q-22q, r = 0.48, *p* = 0.001). However, TERRA levels did not correlate with TA. Ki-67 proliferative indices correlated negatively with Ch-16p and Ch-20q TERRAs (Table 1: r = −0.35, *p* = 0.03; r = −0.42, *p* = 0.01 respectively) suggesting a role related to cell proliferation. Ki67 scores did not correlate with Ch-1q-2q-4q-10q-13q-22q. Ch-16p TERRA also correlated negatively with the Liverpool endometrial steroid quick score (LESQS) for PR (r = −0.40, *p* = 0.02) but the LESQS for other hormone receptors did not show a significant association with TERRA levels (Table 1 and Figure 3). The publicly available “The Cancer Genome Atlas” (TCGA) cohort of uterine cancers dataset suggests high expression of TRF1 gene (*TERF1* RNA) in ECs to be associated with decreased survival (Figure S1). Immunostaining quick-scores for TRF1 protein levels correlated positively with Ch-20q TERRA levels (r = 0.71, *p* = 0.001, Table 1).

**Figure 4:**
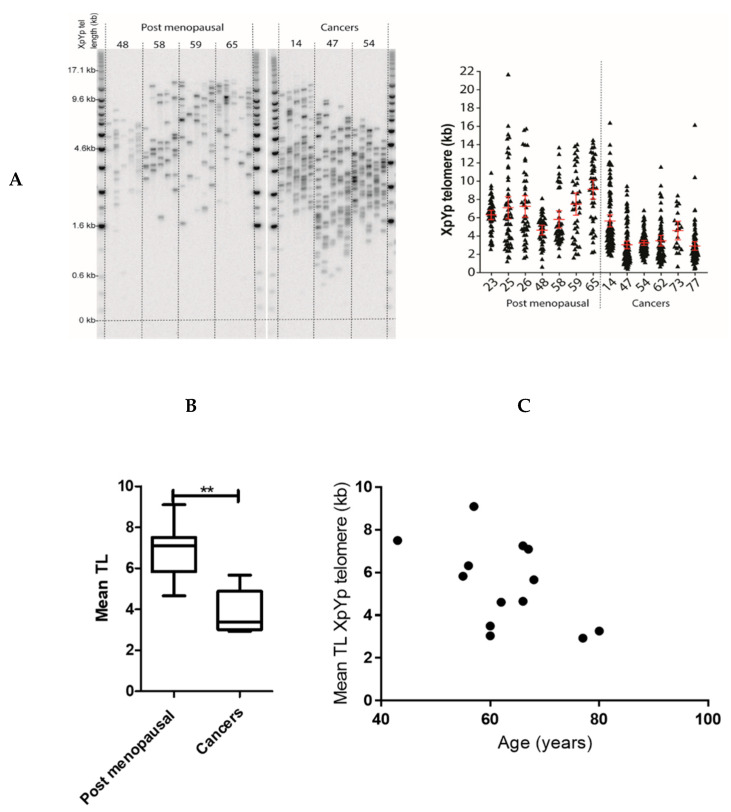
Single telomere length analysis at XpYp telomeres, telomere length distributions in healthy post-menopausal endometrial and endometrial cancer tissue. Representative STELA gel image, together with the distribution of telomere lengths represented as a scatter plot (**A**). Mean telomere lengths in healthy postmenopausal endometrium compared to endometrial cancers. Telomere lengths are significantly shorter in endometrial cancers compared to postmenopausal endometrium (** *p* = 0.002, Mann–Whitney-U test) (**B**). Telomere length correlated with age of the patients, demonstrating no significant association with age and mean telomere lengths (Spearman correlation, r = −0.49, *p* = 0.09) (**C**).

Clear differences were observed between healthy postmenopausal and EC samples in terms of both telomere lengths and variance (SD) of the distribution at the XpYp chromosomes (which is representative of the genome-wide telomere length) (Figure 4A). Postmenopausal endometrial samples displayed considerable telomere length heterogeneity with telomeres at XpYp chromosomes ranging in one sample from 1.3 to 21.7 kb. Consistent with previous reports (reviewed in [5]), ECs displayed shorter telomeres when compared with healthy postmenopausal endometrium (*p* = 0.002, Figure 4B). We found a trend towards a decrease of telomere lengths in older women; however, this difference was not significant (Spearman correlation *r* = −0.49, *p* = 0.09, Figure 4C).

## 3. Discussion

We report here the long non-coding RNA, TERRA, levels in healthy human endometrium from pre- and postmenopausal women and their significant reduction in expression in EC. Although TERRAs have been previously described in human cancers, to our knowledge, the evaluation of TERRA levels in endometrial cancers and their comparison with healthy human endometrial tissues has not been previously reported. The levels of different TERRAs are described in the context of TA, telomere lengths at chromosomes XpYp, immunoscores for ovarian hormone receptors, levels of shelterin proteins TRF1/2 and proliferative indices from the same patient-derived endometrial samples.

We employed a qPCR-based assessment of TERRA levels for its superior quantification quality and reproducibility. Human endometrium is a unique somatic tissue demonstrating dynamic TA, which regulates epithelial proliferation [4]. When we considered the highly proliferative epithelial cells in the proliferative phase endometrium from premenopausal women, which has been associated with high TA and relatively longer average telomere length [22], TERRA levels were remarkably low. The inverse correlation of TERRA levels with epithelial proliferative indices we observed further suggests an association with cellular quiescence for TERRAs in the human endometrium.

Each TERRA had a unique expression pattern, suggesting chromosome-specific regulation of TERRAs. Non-replicative cells with shorter telomeres undergo sub-telomeric rearrangements in yeasts during the quiescent phase of the cell cycle [33], and this further results in increased transcription of non-coding TERRA, agreeing with our data for Ch-20q TERRA in human tissue.

TRF1 and TRF2 are shelterin proteins which protect the chromosomal ends from fusion and initiation of a DDR and they help to regulate TA through a negative feedback mechanism [34]. TRF1 may provide a telomere stabilization function to the short telomeres that we and others have shown in ECs [5]. This may prevent arrest of the cell cycle, thus allowing EC cells to proliferate continuously. The publicly available “The Cancer Genome Atlas” (TCGA) cohort of uterine cancers dataset suggests high expression of TRF1 in ECs to be associated with decreased patient survival (Figure S1). Furthermore, TERRAs can bind to chromosomal locations other than telomeres [35], while the interaction between TERRA and TRF1/2 ensures that TERRA transcripts remain tethered to the telomeric domains [36]. We observed TRF1 protein levels to correlate positively with Ch-20q TERRA levels in EC samples. In contrast, the opposite was observed in benign mouse embryonic stem cells, where an increase in TERRA transcription was observed following abrogation of TRF1 and this may suggest a potential differential interaction between TRF1 and Ch-20q TERRA that may be specific to either human cancers or to EC [20].

We observed high TERRA levels in the proliferatively quiescent PM endometrium, which has low TA levels. In fission yeasts, upregulation of telomere-engaged TERRA can support telomerase-independent telomere maintenance [37]. The high TERRA levels that we report in the TA deficient healthy postmenopausal human endometrium may suggest similar function for human TERRAs, in sustaining telomeres in PM endometrium, preventing genetic instability. A recent in silico study, which examined the TCGA uterine cancers dataset, identified many critical genes associated with telomere maintenance that were previously unknown to contribute to endometrial carcinogenesis and prognosis [25]. However, the intricate relationship between these telomere and telomerase associated genes and proteins with TERRAs is yet to be fully elucidated. Considering the interesting and significant reduction in TERRA levels in EC samples we observed, further examination of this potential interaction is warranted in future studies.

A recent study analyzing endometrioid and serous cancer samples from the TCGA dataset reported EC to be one of the human cancers with the shortest mean telomere lengths among 31 different cancer types [38]. Our data examining individual telomere lengths using STELA at Chromosomes XpYp (which is representative of the genome-wide telomere length) [39] also demonstrate significantly shorter telomeres in EC. Although we were not able to assess the specific telomere lengths at the chromosomes corresponding to the TERRAs tested (Ch16 and 20), collectively, the above data suggest EC to have shorter telomeres. The complex mechanism of TERRA’s role in regulation of telomere length has been previously reviewed and variable mechanisms involving different pathways have been proposed in different physiological conditions. For example, TERRA binding of TLS to G-quadruplex leading to accumulation of H4K20 trimethylation and TERRAs associating with LSD1 and MRE 11 in telomeric 3′G overhang removal leading to lack of protection to chromosomal ends from being recognized as sites of DNA damage, therefore resulting in telomere shortening, were reported [40,41,42,43]. However, our data are the first to evaluate the comparative TERRA levels in healthy and malignant human endometrial tissue, thus we hypothesize that concurrent reduction in TERRA levels and telomere lengths observed in EC may be a global EC related phenomenon. At least, before telomerase re-activation, shorter telomeres may facilitate EC progression due to telomere dysfunction by initiating a “telomere crisis” that drives genomic instability and clonal evolution [44]. We therefore present this interesting observation of reduced TERRA levels in ECs, which co-exist with high TA and short telomeres, thus suggesting a complex, cancer-specific telomere biology in the endometrium. Furthermore, considering the proposed prevention of telomerase accessing telomeres by TERRA [40], the reduction of TERRA may provide a telomere maintenance function and thus proliferative advantage for the ECs. This aspect warrants further studies to ascertain the functional role of TERRA in the endometrium and in EC.

## 4. Materials and Methods

### 4.1. Endometrial Tissue Samples

Endometrial biopsies were obtained from 47 women undergoing gynecological surgery at Liverpool Women’s Hospital. Ethical approval was obtained from the Liverpool Adult Local Research Ethics Committee (LREC; 09/H1005/55, 9 October 2009, NRES Committee North West—Liverpool Central, and 11/H1005/4 6 April 2011, NRES Committee North West—Liverpool Central) and informed written consent was obtained from all patients. Control endometrial samples were collected from women who were not on any hormonal treatment for at least 3 months, with no known endometrial pathology and with regular periods in proliferative phase (*n* = 7), secretory phase (*n* = 9) and postmenopausal (*n* = 7) women, as well as EC samples donated by 24 women with a prior diagnosis of EC (Endometrioid (grade 1 *n* = 6, grade 2 *n* = 7 and grade 3 *n* = 5) and 6 type 2 ECs (serous *n* = 2, carcinosarcoma/malignant mixed Müllerian tumor (MMMT) *n* = 2 and clear cell *n* = 2)) undergoing hysterectomy without receiving any pre-surgical treatment. Patient clinico-pathological and demographic details were retrieved from the clinical notes and electronic databases (Table 2).

### 4.2. RNA Extraction and Real Time-qPCR

RNA was extracted from tissue samples using the Trizol Plus RNA extraction kit (Invitrogen, Life Technologies, Paisley, UK) and quantified using Nanodrop ND-1000 (Thermo Fisher Scientific, Loughborough, UK) as previously described [32]. One microgram of RNA was reverse transcribed with random hexamers using AMV reverse transcriptase (New England Biolabs, Hertfordshire, UK). Two micrograms of cDNA were amplified in triplicate for 40 cycles using iTaq universal SYBR Green supermix and CFX Connect Real Time System (Bio-Rad, Hertfordshire, UK). The specific oligonucleotides used to amplify TERRA transcripts from different chromosome ends (Ch 1q-2q-4q-10q-13p-22p, Ch16p, Ch 20q) and reaction conditions are listed in Table S1. No template and no reverse transcriptase controls were included on each plate and the melt curves were examined to ensure that only a single product was obtained. Relative transcript level was calculated using the ΔΔCT method, normalized to the reference genes, beta actin (*ACTB*) and peptidylprolyl Isomerase A (*PPIA*) using Bio Rad CFX Manager (Bio-Rad, Hertfordshire, UK).

### 4.3. Telomerase Repeat Amplification Protocol (TRAP) Assay

TA was measured using TeloTAGGG TRAP assay (Roche Diagnostics Ltd., Burgess Hill, UK) using 1 µg of lysate [22]. Amplification products after 30 cycles were measured as absorbance at 450 nm in a Fluostar Omega Plate reader (BMG LABTECH, Aylesbury, UK) and presented as arbitrary units (AU).

### 4.4. Single Telomere Length Analysis (STELA)

DNA was extracted using the QIAamp DNA Blood Mini Kit (Qiagen, Manchester, UK). For telomere length analysis at the XpYp telomere, we used the single telomere length analysis (STELA) assay, as previously described [39,44], Genomic DNA was solubilized and diluted in 10 mmol/L Tric-HCl (pH 7.5) to 10 ng/µL. Ten nanograms of DNA were further diluted with 1 µmol/L Telorette2 linker and 1 mM Tris-HCl to 250 pg/µL in a volume of 40 µL. Multiple polymerase chain reactions were conducted to test the DNA sample and were cycled in a Tetrad2 thermocycler (BioRad, Hertfordshire, UK) (22 cycles of 94 °C for 15 s, 65 °C for 30 s, 68 °C for 8 min). DNA fragments were resolved by 0.5% TAE agarose gel electrophoresis and detected by southern hybridization. The hybridized fragments were detected using a phosphorimaging with a Typhoon FLA 9500 phosphoimager (GE healthcare, Chalfont St Giles, UK). The molecular weights of the DNA fragments were calculated using the Phoretix ID quantifier (Nonlinear Dynamics, Newcastle Upon Tyne, UK).

### 4.5. Immunohistochemistry

Formalin-fixed paraffin embedded 3 µm tissue sections were immuno-stained with anti-human TRF1, TRF2, steroid receptors and Ki67 antibodies after antigen retrieval at pH6 as previously described [45]. Antibody sources, concentrations and incubation conditions are detailed in Table S2. Matching isotype (0.5 µg/mL) replaced the primary antibody as a negative control. A specific endometrial tissue sample with positive staining was included as the internal positive control with each staining experiment.

Steroid receptor, TRF1 and TRF2, immunostaining was assessed semi-quantitatively using a four-tiered Liverpool endometrial steroid quick score (LESQS) for steroid receptors and a standard quick score for TRF1 and TRF2 as previously described [45]. The Ki67 proliferative index (PI) was evaluated as the percentage of immunopositive cells of any intensity. Epithelial and stromal cell staining was scored separately based on morphological criteria in postmenopausal and malignant endometrium and stratum basalis of healthy proliferative phase and secretory phase endometrium by two independent observers. Discrepancies between the two observers were resolved by re-evaluating the samples together and agreeing on a final score.

### 4.6. Analysis of TCGA Dataset

The publicly available TCGA cohort of uterine cancers included data for RNA levels for *TERF1* and was interrogated using Illumina’s Base Space Cohort Analyzer application (BSCA) (https://www.illumina.com/informatics/research/biological-data-interpretation/nextbio.html; Illumina, San Diego, CA, USA) [46].

### 4.7. Statistical Analysis

Gene expression data were analyzed using GraphPad Prism software version 5 (San Diego, CA, USA) using non-parametric tests (Mann–Whitney U test or Spearman correlation as appropriate, not-assuming Gaussian distribution). The criterion for significance was *p* ≤ 0.05.

## 5. Conclusions

TERRAs seem to be dynamically expressed in the healthy human endometrium, and significantly lower TERRA levels are found in ECs. Since our study showed TERRA levels to be inversely related to endometrial epithelial cellular proliferative indices, further studies are required to determine whether these levels can be successfully modified and tailored as a therapeutic target in endometrial proliferative conditions.

## Figures and Tables

**Table 1:** Correlation of chromosomes 1q-2q-4q-10q-13q-22q (TERRA 1), TERRA 16 TERRA 20, LESQS scores for AR, PR, ERα and ERβ and immuno-scores for Ki67, TRF1, TRF2 and TA measured by TRAP assay in all endometrial samples. Values in **bold letters** show significant correlations

	TERRA1		TERRA16		TERRA20	
	r	*p*	r	*p*	r	*p*
**TERRA1**			**0.4885**	**0.0008**	**0.4841**	**0.0009**
**TERRA16**	**0.4885**	**0.0008**			**0.7792**	**<0.0001**
**TERRA20**	**0.4841**	**0.0009**	**0.7792**	**<0.0001**		
**AR**	−0.08156	0.6465	−0.2828	0.1051	−0.1727	0.3288
**PR**	−0.1068	0.5543	**−0.3997**	**0.0212**	−0.2094	0.2423
**ERα**	−0.0579	0.7411	0.1462	0.4019	0.198	0.2543
**ERβ**	0.01555	0.9293	0.1325	0.4479	0.08311	0.635
**Ki67**	−0.147	0.3854	**−0.3487**	**0.0319**	**−0.4156**	**0.0095**
**TRF1**	0.4547	0.0505	0.4414	0.0585	**0.7083**	**0.0007**
**TRF2**	0.08588	0.8432	0.4269	0.2499	0.4809	0.1938
**TRAP**	−0.1997	0.2292	−0.251	0.1285	−0.09328	0.5775

**Table 2:** Demographic features of study groups.

Study Groups (*n*)	* Age (years)	* BMI (kg /m^2^)
Proliferative phase (7)	43 (32–57)	27.8 (22–40.5)
Secretory phase (9)	41 (21–47)	22.6 (18.9–31.6)
Postmenopausal (7)	62 (52–85)	24.3 (20–39.6)
Total Endometrial cancer pts (24)	67 (37–80)	30 (23.9–54.4)
Endometrioid Grade 1 (6/24)	61 (46–73)	37.8 (28.3–46.1)
Endometrioid Grade 2 (7/24)	60 (37–77)	28.9 (25.8–54.4)
Endometrioid Grade 3 (5/24)	68 (60–80)	29.8 (23.9–42.7)
Malignant Mixed Mullerian Tumor (2/24)	72.5 (65–80)	28.6 (24.2–32.9)
Clear Cell Carcinoma (2/24)	71.5 (61–82)	28.4 (26.6–30.1)
Serous Carcinoma (2/24)	73 (68–78)	32.7 (NK **-32.7)

* Data expressed as median (range). ** BMI not known (NK) for 1 patient.

## Supplementary Materials

Supplementary Materials can be found at https://www.mdpi.com/1422-0067/21/22/8686/s1. Figure S1: The Cancer Genome Atlas (TCGA) RNA Sequencing Data (endometrioid and serous cancers) for shelterin protein TRF1, Table S1: Oligonucleotides used for qPCR amplification, Table S2: Primary antibodies and conditions for IHC.
