# Supplementary material for: Human Endometrial Carcinogenesis Is Associated with Significant Reduction in Long Non-Coding RNA, TERRA
Source: Int J Mol Sci. 2020 Nov 18;21(22):8686. doi: 10.3390/ijms21228686 (PMC7698627; doi:10.3390/ijms21228686)

Supplementary table 1. Primer sequences used for qPCR amplification

Primer	Sequence	PCR conditions	Primer efficiency	References
TERRA Ch 1q-2q-4q-10q-13q-22q	F:5'GAATCCTGCGCACCGAGAT<br>R:5'CTGCACTTGAACCCTGCAATAC	95°C, 10 min<br>followed by 40 cycles of<br>98°C, 5s<br>95°C, 10s<br>60°C, 30s	101.1%<br>R^2=0.995	(Diman, et al., 2016)
TERRA Ch16p	F: 5'TGT GTT TCA ACG CTG CAA CTG<br>R: 5'AGT TAG AAC GGT TCA GTG TG		95.1%<br>R^2=0.991	(Wang, et al., 2015)
TERRA Ch20q	F:5'GAAGTTGCTGGGTTCTATGG<br>R:5'ATGGTGCAGACACTGTGG		105.2%<br>R^2=0.986	(Montero, et al., 2016)
ACTB	F:5'TGTACGCCAACACAGTGCTG-3<br>R:5'GCTGGAAGGTGGACAGCGA-3		94.6%<br>R^2=0.999	(Wang, Zhao and Lu, 2015)
PPIA	F:5'- AGACAAGGTCCCAAAGAC-3<br>R:5'- ACCACCCTGACACATAAA-3	95°C, 2 min<br>followed by 40 cycles of 95°C 5s, 60°C 30s	100.1%<br>R^2=0.998	(Jacob, et al., 2013)

References :

Diman A, Boros J, Poulain F, Rodriguez J, Purnelle M, Episkopou H, Bertrand L, Francaux M, Deldicque L, Decottignies A. Nuclear respiratory factor 1 and endurance exercise promote human telomere transcription. *Sci Adv* 2016;2.

Jacob F, Guertler R, Naim S, Nixdorf S, Fedier A, Hacker NF, Heinzelmann-Schwarz V. Careful Selection of Reference Genes Is Required for Reliable Performance of RT-qPCR in Human Normal and Cancer Cell Lines. *Plos One* 2013;8.

Montero JJ, de Silanes IL, Grana O, Blasco MA. Telomeric RNAs are essential to maintain telomeres. *Nature communications* 2016;7.

Wang CQ, Zhao L, Lu SM. Role of TERRA in the Regulation of Telomere Length. *Int J Biol Sci* 2015;11: 316-323.

Supplementary Table 2. Primary antibodies and conditions for IHC

Primary Antibody	Type	Clone	Supplier		Dilution	Incubation Time	Conditions
				HIAR* (min)		Time (hour)	Temp (°C)
ERα	Monoclonal	6F11	NovoCastra[1]	2	1:50	2	18
ERβ	Monoclonal	PPG5/10	Abcam[2]	2	1:50	20	4
PR	Monoclonal	PgR 636	DAKO[3]	2	1:1000	1	18
AR	Monoclonal	AR441	DAKO[3]	2	1:75	20	4
Ki67	Monoclonal	MM1	NovoCastra[1]	2	1:200	20	4
TRF1	Monoclonal	MM1	Santa Cruz[4]	2	1:50	20	4
TRF2	Monoclonal	MM1	Santa Cruz[4]	2	1:200	20	4

*Heat induced antigen retrieval by pressure cooking in citrate buffer pH 6. [1]Dallas, Texas, USA;

[1] Newcastle, UK;2 Cambridge, UK; [3] Ely, Cambridgeshire,UK; 4 Heidelburg, Germany

Supplementary Figure 1

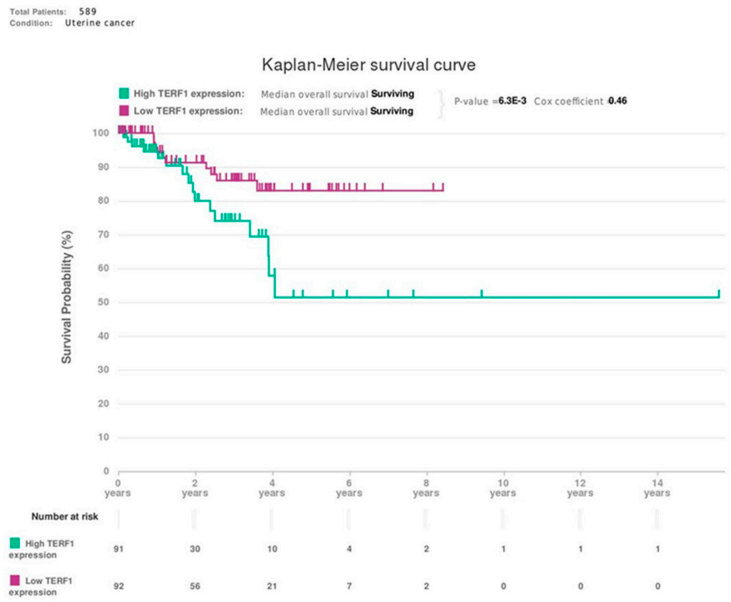

Supplement: Supplementary file 1 [file ijms-21-08686-s001.pdf]
